# Identification of novel protein biomarkers and therapeutic targets for ankylosing spondylitis using human circulating plasma proteomics and genome analysis

**DOI:** 10.1007/s00216-024-05521-4

**Published:** 2024-09-10

**Authors:** Zhongxian Zhou, Chong Liu, Sitan Feng, Jiarui Chen, Tianyou Chen, Jichong Zhu, Shaofeng Wu, Chenxing Zhou, Chengqian Huang, Jiang Xue, Xiaopeng Qin, Xinli Zhan

**Affiliations:** Spine Surgery, The First Affiliated Hospital of Guangxi Medical University, No. 6 Shuangyong Road, Qingxiu District, Nanning, 530021 Guangxi People’s Republic of China

**Keywords:** Ankylosing spondylitis, Mendelian randomization, scRNA-seq, Novel protein biomarkers

## Abstract

The proteome serves as the primary basis for identifying targets for treatment. This study conducted proteomic range two-sample Mendelian randomization (MR) analysis to pinpoint potential protein markers and treatment targets for ankylosing spondylitis (AS). A total of 4907 data points on circulating protein expression were collected from a large-scale protein quantitative trait locus investigation involving 35,559 individuals. Using data from a Finnish study on AS as the outcome, the dataset comprised 166,144 individuals of European ancestry (1462 cases and 164,682 controls), and causal relationships were determined through bidirectional Mendelian randomization of two samples. Proteins were further validated and identified through single-cell expression analysis, certain cells showing enriched expression levels were detected, and possible treatment targets were optimized. Increased HERC5 expression predicted by genes was related to increased AS risk, whereas the expression of the remaining five circulating proteins, AIF1, CREB3L4, MLN, MRPL55, and SPAG11B, was negatively correlated with AS risk. For each increase in gene-predicted protein levels, the ORs of AS were 2.11 (95% CI 1.44–3.09) for HERC5, 0.14 (95% CI 0.05–0.41) for AIF1, 0.48 (95% CI 0.34–0.68) for CREB3L4, 0.54 (95% CI 0.42–0.68) for MLN, 0.23 (95% CI 0.13–0.38) for MRPL55, and 0.26 (95% CI 0.17–0.39) for SPAG11B. The hypothesis of a reverse causal relationship between these six circulating proteins and AS is not supported. Three of the six protein-coding genes were expressed in both the AS and healthy control groups, while CREB3L4, MLN, and SPAG11B were not detected. Increased levels of HERC5 predicted by genes are related to increased AS risk, whereas the levels of the remaining five circulating proteins, AIF1, CREB3L4, MLN, MRPL55, and SPAG11B, negatively correlate with AS risk. HERC5, AIF1, and MRPL55 are potential therapeutic targets for AS. This study advanced the field by employing a novel combination of proteomic range two-sample MR analysis and single-cell expression analysis to identify potential protein markers and therapeutic targets for AS. This approach enabled a comprehensive understanding of the causal relationships between circulating proteins and AS, which has not been extensively explored in previous studies.

**Graphical Abstract:**

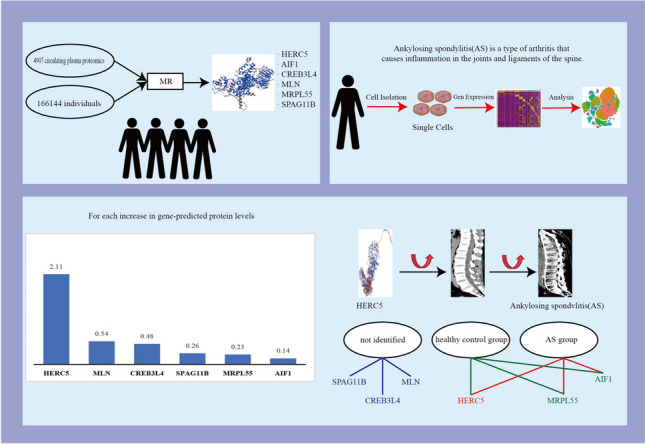

**Supplementary Information:**

The online version contains supplementary material available at 10.1007/s00216-024-05521-4.

## Introduction

Ankylosing spondylitis (AS) is a chronic progressive inflammatory disorder that primarily affects the axial skeleton, leading to characteristic inflammatory lower back pain and severe structural and functional impairments [1, 2]. Reports indicate that the global incidence of AS ranges from 0.07 to 0.32%, with a male prevalence 2–3 times higher than that of females [3, 4]. AS is a challenging disorder characterized by high incidence, significant damage risk, and substantial nursing costs, often referred to as the “three highs.” These challenges are likely exacerbated by a limited understanding of AS pathogenesis and progression. Recognized risk factors for AS include genetic predisposition, infections, mechanical stress, gut microbiota, sex, environmental, and lifestyle factors [5]. Recent studies suggest a potential link between HLA-B27, AS, and intestinal immune insufficiency [6–8]. However, the precise pathogenic mechanisms of AS remain unknown, and effective treatments are still lacking. Therefore, it is crucial to further investigate the pathogenic mechanisms of AS to identify new therapeutic directions and improve treatment efficacy.

Recent advancements in proteomics have provided valuable insights into the molecular mechanisms underlying AS [9, 10]. Proteomics, the large-scale study of proteins, particularly their structures and functions, has been utilized to identify protein biomarkers and elucidate pathways involved in AS. For instance, Wang et al. conducted a proteomic analysis that identified several proteins involved in immune response and inflammation that were differentially expressed in AS patients compared to healthy controls [6]. This study highlighted the potential role of these proteins in AS pathogenesis and their value as biomarkers for disease diagnosis and progression. Additionally, another significant study by Bowden et al. utilized quantitative proteomics to profile the proteome of AS patient samples and discovered dysregulation in pathways related to cytokine signaling and immune regulation [10]. This research not only identified potential therapeutic targets but also underscored the complex nature of AS, involving multiple pathways and regulatory networks.

Despite these advancements, few studies have explored the causal relationships between plasma circulating proteins and AS. Mendelian randomization (MR) offers a promising approach to address this gap. MR utilizes genetic variation as an instrumental variable for exposure (e.g., circulating proteins), enhancing causal inference by minimizing confounding due to the random assortment of genetic variants during conception and independence from environmental and self-selection factors. Cyclic proteins, which are pivotal regulatory elements in molecular pathways, have consistently been considered potential drug targets [11, 12]. Recent advances in the genetic study of circulating protein expression across multiple samples provided an opportunity for an in-depth examination of the causal relationship between circulating proteins and AS. Single-cell sequencing technology offers insights into gene expression heterogeneity between cells, providing detailed information at the cellular level.

In this study, a novel approach was employed by combining proteomic MR analysis with single-cell transcriptome analysis to investigate the causal relationship between circulating proteins and AS. This innovative methodology aimed to reveal potential targets for anti-AS treatment, offering a new direction for developing efficient therapeutic agents and enhancing AS treatment efficacy. This study addressed a significant gap in the current understanding of the causal relationships between circulating plasma proteins and AS. Previous studies have primarily concentrated on the identification of dysregulated proteins in AS without establishing causality. In contrast to earlier studies that have identified dysregulated proteins in AS without establishing causality, this study integrated two-sample MR analysis with single-cell RNA sequencing (scRNA-seq) validation; this study presented a novel methodology for identifying protein biomarkers and therapeutic targets, thereby providing a more robust framework for understanding AS pathogenesis and identifying potential treatments.

## Methods

### Research design and ethics

**Fig. 1:**
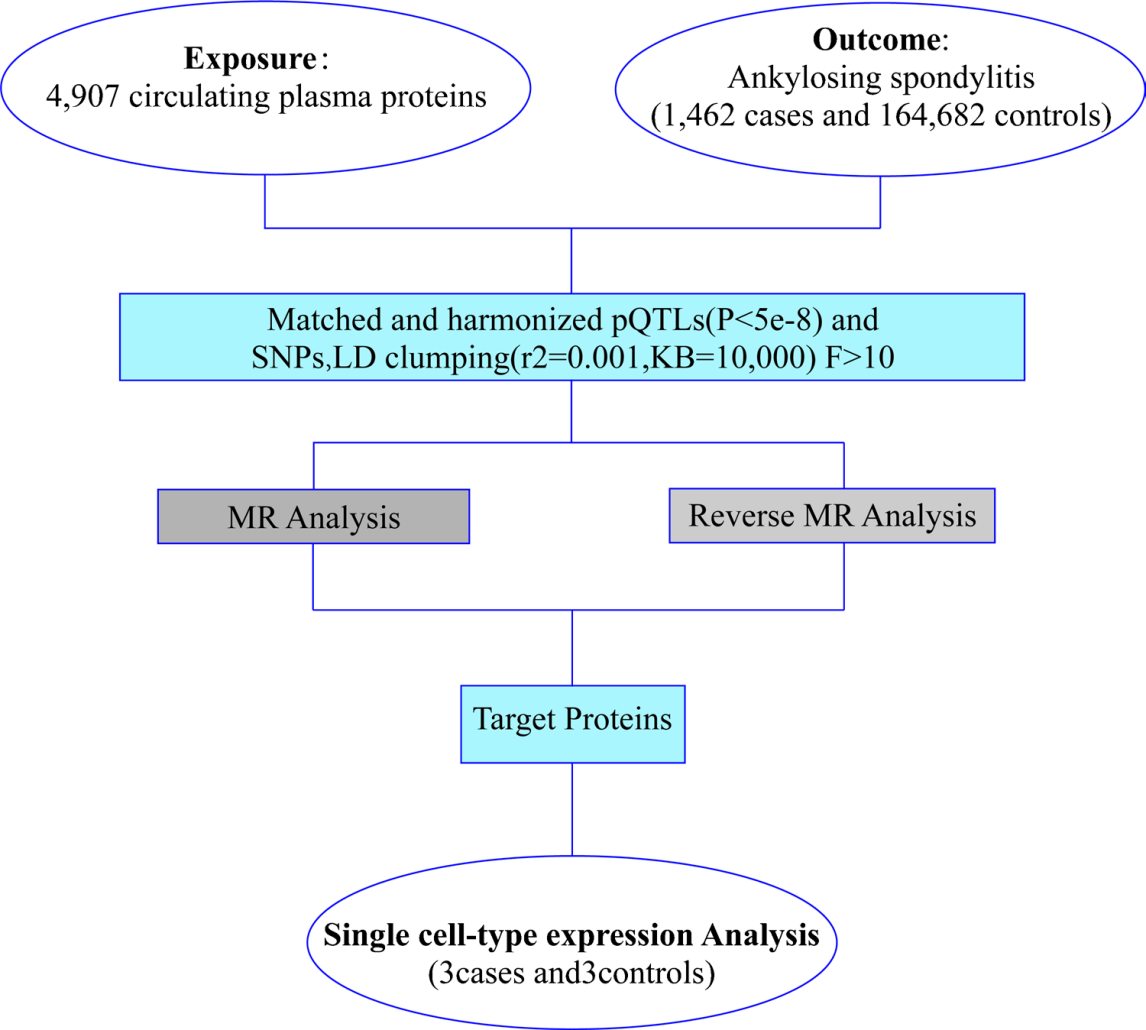
Flowchart of the study design

Figure 1 illustrates our research methodology. This study was conducted utilizing a comprehensive dataset of blood protein groups from genome-wide association studies sourced from publicly available data (https://www.decode.com/summarydata/) [7], as well as GWAS data associated with AS investigations from Finland (https://gwas.mrcieu.ac.uk/datasets/finn-b-M13_ANKYLOSPON/). The database includes 166,144 individuals of European ancestry, comprising 1462 AS patients and 164,682 controls. AS was diagnosed through the revised New York (mNY) criteria [8]. Through consultation with the FinnGen Consortium, we confirmed that the selection of AS was based on the International Classification of Diseases-10 (ICD-10) diagnostic code (M45-M49). The appropriate ethical review committee approved the enrolled articles.

### Proteomic data

Statistics for genetic associations with 4907 circulating proteins at the abstract level were obtained from a large protein quantitative trait locus (pQTL) study of 35,559 Icelanders [7]. After that, we conducted proteomic analysis through a multiplex, modified aptamer-based binding assay (SOMAscan version 4). Normal changes in protein levels reversed with age and sex. In addition, rank-inverse normal transformation was performed to normalize residuals, with normalized data being used as phenotypes for genome-wide association analysis in the BOLT-LMM linear hybrid model. More GWAS data were obtained from the original article [9]. Existing studies have shown that the PQTLS protein reaches genome-wide significance (*P* < 5e-8) in two-sample MR.

### Results data source

The aggregate GWAS data included data on the effects of nucleotide polymorphisms (SNPs) on phenotypes. GWAS data related to AS were obtained from a study in Finland. The database included 166,144 subjects of European descent (1462 cases and 164,682 controls) (https://gwas.mrcieu.ac.uk/datasets/finn-b-M13_ANKYLOSPON/). For this MR analysis, we limited AS patients to those of European ancestry to minimize possible population heterogeneity-induced bias [10].

### Selection of instrumental variables

Significant SNPs closely associated with AS were identified based on a genome-wide significance threshold of < 5 × 10–8. To ensure independence among IVs, SNPs exhibiting linkage disequilibrium (LD) were eliminated (*r*2 = 0.001, KB = 10,000). The association strength of SNPs with exposure was estimated using the *F*-statistic (*F* = *b*2/SD2, where *b* represents the magnitude of the effect of SNP on exposure, and SD is the standard deviation) (10). We eliminated genetic variations whose *F* values were < 10 (had a low capacity for explaining exposure) [11] to avoid bias in the effect estimation.

### MR analysis

Three core hypotheses of MR research were assessed [12]: (i) strong and robust correlation between genetic IVs and AS (association hypothesis); (ii) independence of gene IVs from confounders affecting the exposure-outcome relation (independence hypothesis); and (iii) exclusive effect of gene IVs on AS through circulating proteins [13]. A series of MR research methods were employed to examine potential pleiotropy to validate the 2nd and 3rd MR hypotheses. Horizontal pleiotropy occurs when genetic variation influences the exposure pathway and other traits or directly impacts the outcome [14]. We conducted two-sample MR analysis based on protein index SNPs and estimation of the protein-to-prognosis ratio (ORs) and relevant confidence intervals (CIs).

### Statistical analysis

Inverse variance weighting (IVW), MR-Egger regression, simple mode, weighted mode, the weighted median method, MR multiple effect residuals, and outliers (MR-PRESSO) were employed for causal effect estimation and evaluation of MR analysis reliability and stability. Heterogeneity analysis assessed heterogeneity between IVs, with a threshold of *P* < 0.05 indicating possible heterogeneity [15]. The MR-Egger intercept is employed for horizontal pleiotropy assessment, wherein a y-intercept of 0 signifies no horizontal pleiotropy effect. Moreover, horizontal pleiotropy can be assessed via the MR-PRESSO global test, contributing to the estimation of global heterogeneity for detecting pleiotropy [12, 14], with a *P* value less than 0.05 indicating pleiotropy among the chosen IVs. Finally, to comprehensively evaluate the robustness of the MR results, we utilized the leave-one-out method to assess whether excluding one SNP would impact the overall effect of the remaining SNPs. Notably, the results obtained by IVW analysis exhibit statistical significance, whereas additional complementary methods do not, suggesting no apparent pleiotropy. When horizontal pleiotropy of IVs is not present, IVW has the highest statistical power, so it is used as the primary analysis method. Other MR methods take into account different types of genetic pleiotropy and are based on potentially different assumptions that are made to check the robustness of the results. It may be considered a favorable outcome if other complementary methods show consistent directional values. In this study, we considered a threshold of *P* < 0.05 to indicate a statistically significant causal relationship. The aforementioned statistical analysis was conducted using the TwoSampleMR package of R (version 4.3.2).

### Reverse MR analysis

To explore whether AS has a causal effect on the detected circulating proteins, reverse MR analysis (with AS being an exposure and detected circulating proteins being an outcome) was conducted. The analysis and statistical methods used are described above.

### Single-cell‑type expression analysis

scRNA-seq data were collected from AS patients (*n* = 3) and healthy controls (*n* = 3) at the Spinal Surgery Department of the First Affiliated Hospital of Guangxi Medical University. The samples used for single-cell sequencing were obtained from the spinal bone marrow blood of patients who underwent surgery and fully met the modified New York AS criteria. We further validated the plasma protein levels of target genes, showing possible causal effects on AS in specific cells [16, 17]. The RNA-seq data of 57,684 AS patients were obtained. We first used the “Seurat” software package [18] to preprocess and convert the raw single-cell RNA-seq data. Genes whose count was less than 3 and whose unique feature count was less than 50 in a single cell were removed from the cells. Then, the normalizeData function was adopted to normalize and scale the data. Cell types were labeled with the “SingleR” package [19]. To test whether the detected AS pathogenic protein-coding genes were highly expressed in specific cell types of AS patients, we conducted differential expression analysis using the FindAllMarkers command to compare gene expression levels among different cell types. Genes whose mean log2-fold change (log2FC) was > 1 and adjusted false discovery rate (FDR) *P* value was < 0.05 were considered to be enriched in cells.

## Results

### A full proteome MR analysis identified six circulating proteins in AS

**Fig. 2:**
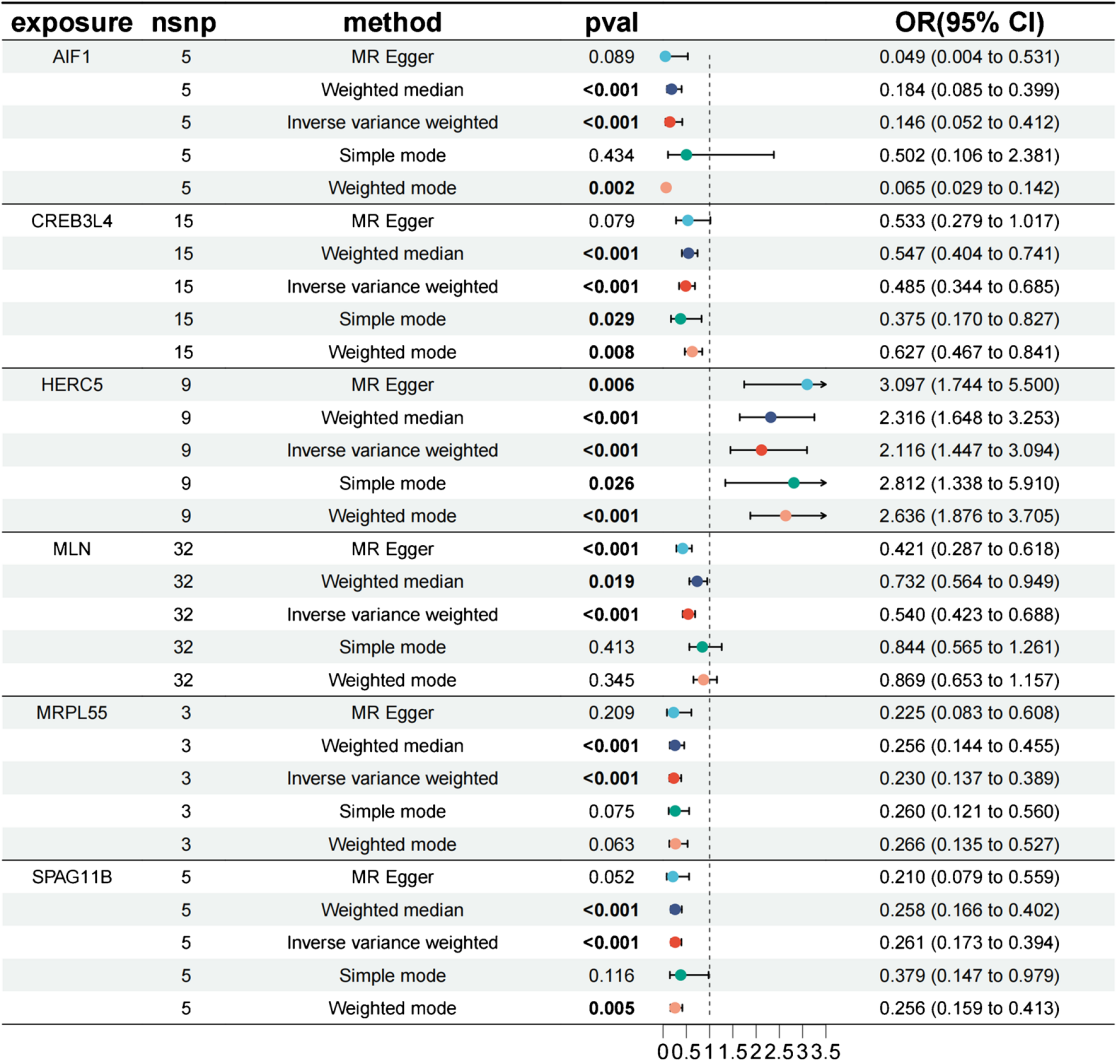
The MR estimation of the causal effect of exposure (circulating proteins) on outcomes (ankylosing spondylitis) obtained through different MR methods is represented in the forest plot (MR, Mendelian randomization; OR, odds ratio; CI, confidence interval)

**Fig. 3:**
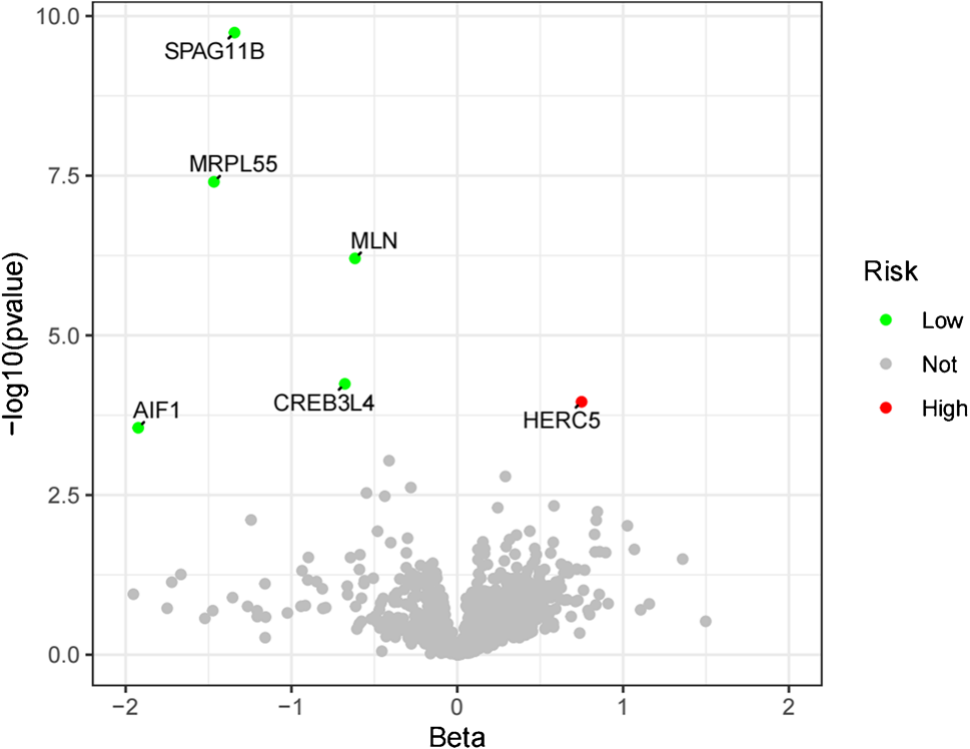
Volcano plot showing the results of MR of the circulating proteins

Initially, we screened SNPs associated with circulating proteins as IVs, with a significance level of *P* < 5 × 10–8. Among these, 25,604 SNPs related to AS were identified (Supplementary Table 1). Subsequently, each protein was analyzed using five methods for two-sample MR analysis (Supplementary Table 2). By employing the IVW method, the MR results were further refined (*P* < 0.05, FDR < 0.2), revealing six proteins associated with AS without pleiotropy (*P* > 0.05) (Fig. 2, Supplementary Table 3). According to multiple tests and corrections, higher levels of HERC5 predicted by genes were linked to increased AS risk, while the levels of the remaining five circulating proteins (AIF1, CREB3L4, MLN, MRPL55, and SPAG11B) were negatively correlated with AS risk (Fig. 3). For each increase in gene-predicted protein levels, the ORs of AS were 2.11 (95% CI 1.44–3.09) for HERC5, 0.14 (95% CI 0.05–0.41) for AIF1, 0.48 (95% CI 0.34–0.68) for CREB3L4, 0.54 (95% CI 0.42–0.68) for MLN, 0.23 (95% CI 0.13–0.38) for MRPL55, and 0.26 (95% CI 0.17–0.39) for SPAG11B. No pleiotropy was found (*P* > 0.05) (Supplementary Table 4). A sensitivity analysis was subsequently conducted to confirm that our MR results were robust (Supplementary Figs. 1–6).

### Reverse MR analysis

To explore the reverse causal relationship between the six identified circulating proteins and AS, we conducted reverse MR analysis. We applied the inverse variance weighting (IVW) method, MR-Egger regression, the weighted median method, the simple mode, and the weighted mode for cyclic analysis of these six proteins (Supplementary Table 5). The MR estimation results from the IVW method indicated that AIF1 (*P* = 0.36, OR = 1.00, 95% CI 0.99–1.01), MLN (*P* = 0.40, OR = 1.01, 95% CI 0.98–1.03), SPAG11B (*P* = 0.61, OR = 0.99, 95% CI 0.98–1.01), MRPL55 (*P* = 0.09, OR = 1.01, 95% CI 0.99–1.02), CREB3L4 (*P* = 0.15, OR = 0.98, 95% CI 0.961.00), and HERC5 (*P* = 0.60, OR = 1.00, 95% CI 0.98–1.01) had no significant effects on AS risk. Estimates of MR causal effects using the other four supplementary approaches did not reach statistical significance (all *P* > 0.05). Therefore, the hypothesis of a reverse causal relationship between these six circulating proteins and AS is not supported. Sensitivity analysis using MR-PRESSO did not detect SNPs associated with pleiotropy (all *P* > 0.05). Additionally, we observed heterogeneity and horizontal pleiotropy (*P* > 0.05) among the genetic tools for five proteins (AIF1, CREB3L4, MLN, MRPL55, and SPAG11B) (Supplementary Tables 6 and 7). The heterogeneity of the HERC5 genetic tool was *P* < 0.05, but its IVW pattern was *P* > 0.05, while pleiotropy was *P* > 0.05, considering differences in the data from different studies.

### Single-cell data validation

**Fig. 4:**
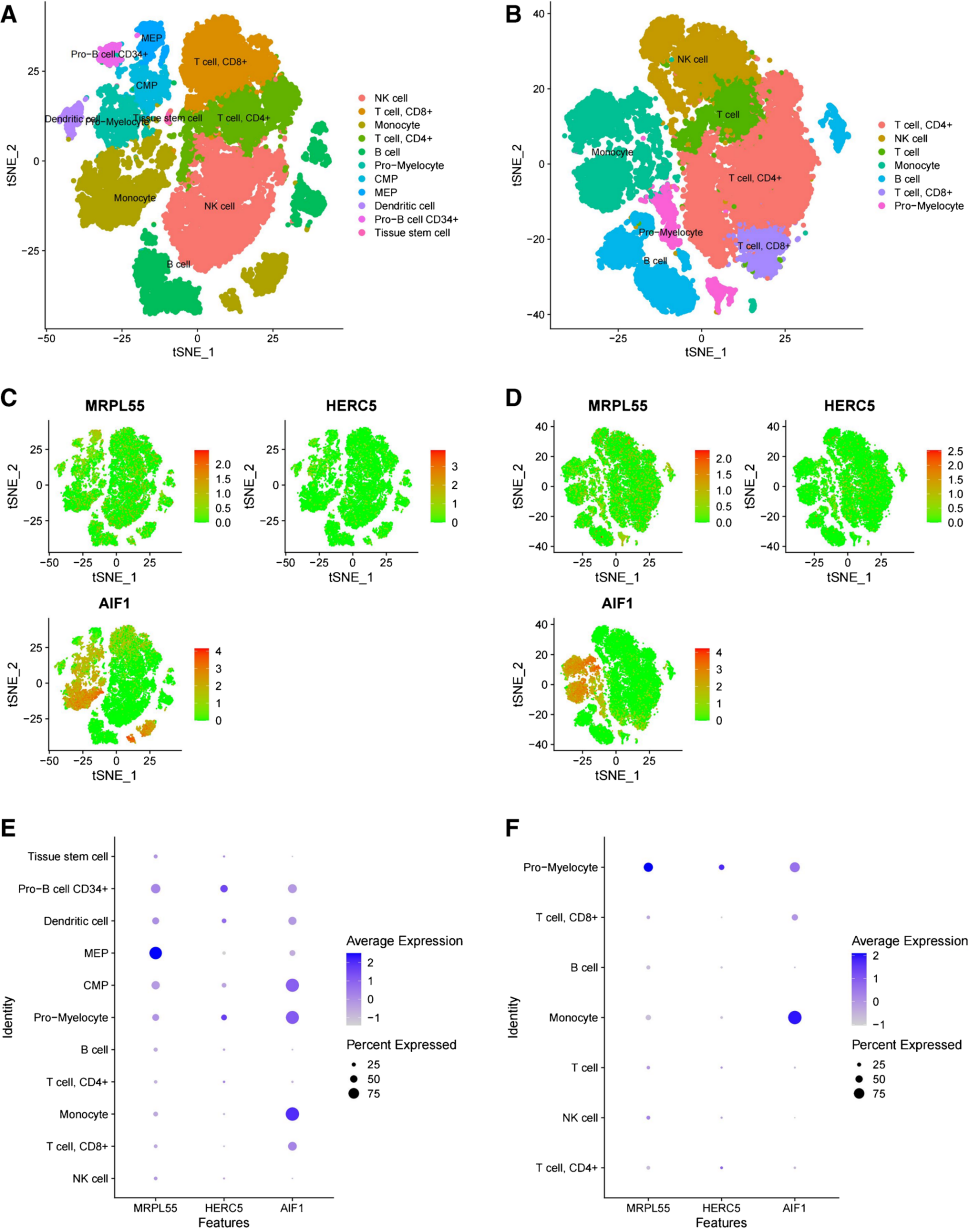
Single-cell sequencing was used to identify the protein-coding genes of the AS group and the healthy group and to verify the Mendelian randomization results of the proteome. **A** A total of 19 cell clusters and 11 cell types were identified in the AS group. **B** A total of 19 cell clusters and 11 cell types were identified in the healthy group. The expression of protein-coding genes in each cluster of the AS group (**C**, **E**) and healthy group (**D**, **F**)

To investigate whether six protein genes were enriched in specific cells of AS patients, single-cell RNA sequencing was performed in the AS group and the healthy group. The AS group cells were categorized into 19 clusters and divided into 11 cell types (NK cells, T cells, CD8 + cells, monocells, T cells, CD4 + cells, B cells, promyelocytes, CMPs, MEPs, dendritic cells, Pro-B cells, CD34 + cells, and tissue stem cells) (Fig. 4A). The cells in the healthy group were also categorized into 19 clusters and divided into 7 cell types (T cells, CD4 + cells, NK cells, NK cells, monocytes, B cells, T cells, CD8 + cells, and promyelocytes) (Fig. 4B). Three out of the six protein-coding genes were expressed in both sets of data, while CREB3L4, MLN, and SPAG11B were not expressed (Fig. 4C, D). The bubble plot shows the single-cell expression of three coding genes of each cell type, with an average Log2FC > 1 and FDR < 0.05 (Fig. 4E, F). In the AS group, MRPL55 was mainly expressed on MEPs, CD34 + Pro-B cells, CMPs, dendritic cells, and promyelocytes. AIF1 was mainly enriched in monocells, CMP, promyelocytes, Pro-B cells CD34 + , T cells, and CD8 + cells, while HERC5 was upregulated in Pro-B cells CD34 + , promyelocytes, and dendritic cells. In the healthy group, MRPL55 was mainly expressed in promyelocytes, AIF1 was mainly enriched in monocytes and promyelocytes, and HERC5 was mainly enriched in promyelocytes.

## Discussion

In the present work, two-sample MR analysis was conducted to determine the causal relationship between 4907 circulating plasma proteins and the risk of AS, and the causal relationships between genetic circulating plasma proteins and AS were evaluated through bidirectional evaluation. SNPs serve as instrumental variables and remain unaffected by confounding factors or reverse causal relationships [20]. Six protein markers were identified through proteomic MR analysis. Elevated levels of circulating HERC5 predicted by genes were associated with increased AS risk, while elevated levels of circulating AIF1, CREB3L4, MLN, MRPL55, and SPAG11B predicted by genes were associated with decreased AS risk. Reverse MR analysis demonstrated the causal effects of these six protein biomarkers on AS, providing genetic evidence supporting the causal relationship between circulating plasma proteins and AS. Taking into account ethnic differences, we tested the expression of circulating proteins in our countrymen by sequencing 3V3 single-cell samples. The data showed that protein-coding genes were differentially expressed between the AS and the healthy groups, suggesting that these genes play a crucial role in recognizing and treating AS.

Based on the current research, HERC5 has emerged as a promising therapeutic target for AS, as supported by robust evidence. Hect domain and RCC1-like domain-containing protein 5 (HERC5) is a crucial ubiquitin ligase belonging to the E3 family of ubiquitin ligases [21] that regulates ubiquitination within cells and influences diverse cellular functions and signaling pathways [22]. HERC5 exhibits ubiquitin ligase activity, enabling it to label target proteins for ubiquitination by binding to specific lysine residues, which is a vital modification process governing various cellular processes [23]. As observed in *Mycobacterium tuberculosis* infection, HERC5 modulates immune responses through ubiquitination. In this case, it regulates macrophages and impacts immune responses [22]. Furthermore, HERC5 plays a role in regulating cellular signaling pathways. It influences abnormal breast cancer cell growth, invasion, and gene expression levels, thereby contributing to breast cancer development [24, 25]. Additionally, allograft inflammatory factor 1 (AIF1) has emerged as another significant immune- and inflammation-related protein that participates in diverse biological processes through various pathways, including immune and inflammatory responses and cellular signaling [26, 27]. AIF1, ankylosing spondylitis characterized by a systemic inflammatory response, also provides us with a research direction on how it regulates the inflammatory response of the synovium and ligament and the involvement of related inflammatory mediators. AIF1 induction by TNF-α stimulation promotes macrophage recruitment and liver inflammation, affecting the liver microenvironment and potentially contributing to liver cancer development [28]. Moreover, AIF1 serves as a potential tumor marker involved in tumor immune cell infiltration and promoting tumor progression [26]. AlF1 can activate the NF-KB pathway and the expression of relevant target genes related to inflammation, cell apoptosis, and the stress response [29]. Moreover, AlF1 is also involved in the pathogenesis of rheumatoid arthritis (RA) and is an important cytokine [30]. MRPL55 (mitochondrial ribosomal protein L55) is a component of mitochondrial ribosomes and is involved in mitochondrial protein synthesis. An increase in the MRPL55 level in hepatocellular carcinoma can serve as a potential diagnostic marker [31]. MRPL55 can also be used as an observational indicator for the prognosis of ovarian cancer patients [32]. cAMP response element binding protein 3-like 4 (CREB3L4) is a transcription factor that belongs to the CREB3 family. The CREB3L4 gene encodes a protein known as CREB3L4 protein that is expressed in humans. Its cellular function primarily involves signal transduction at the endoplasmic reticulum membrane and regulation of the cellular stress response [33]. Notably, CREB3L4 contributes to hepatocellular carcinoma development by promoting the RHEB-mTORC1 pathway and reducing sensitivity to chemotherapy drugs [34]. Constitutive overexpression of CREB3L4 inhibits adipocyte differentiation, whereas its downregulation promotes the differentiation of preadipocytes into mature adipocytes. CREB3L4 is a potential therapeutic target for treating obesity and metabolic syndrome [35]. We found that CREB3L4 is highly upregulated in a specific subtype of triple-negative breast cancer, the intraluminal androgen receptor (LAR) subtype, and can be used as a potential therapeutic target [32]. Sperm-associated antigen 11B (SPAG11B) can be detected mostly within the testes and has a critical effect on the normal function of the reproductive system. SPAG11B, a testicular-specific protein, has absolute or almost absolute specificity and sensitivity for observing dysfunction in supporting cells or germ cells [36]. Prior research highlighted innovative therapeutic protein targets for AS and assessed the potential adverse effects of druggable proteins. A positive genetic correlation was identified between the predicted plasma concentrations of six proteins and an elevated susceptibility to AS, while two proteins exhibited a negative correlation with AS risk (Pfdr < 0.05). Among these eight plasma proteins, colocalization analysis revealed that AIF1, TNF, FKBPL, AGER, ALDH5A1, and ACOT13 share significant genetic variation with AS (PPH3 + PPH4 > 0.8). This suggests that these proteins could serve as direct therapeutic targets for AS intervention [37]. We also observed that the presence of one protein (MLN) is strongly supported by genetic evidence. Still, there are relatively few reports on the MLN protein, and further research is needed. However, we cannot eliminate the possibility of heterogeneity-induced associations. Therefore, based on the validation results of the single-cell data, HERC5, AIF1, and MRPL55 were identified as potential therapeutic targets for AS.

The present study offers several advantages, as we comprehensively explored the relationship between circulating plasma proteins and atherosclerosis risk using a two-stage proteomic MR system. Two-sample MR provides substantial benefits due to its extensive sample size, broad proteomic coverage, and minimized confounding bias and reverse causality risk. Rigorous analysis incorporating multiple validity, heterogeneity, and sensitivity assessments ensured the reliability of our research findings [38]. Moreover, integrating single-cell data provided valuable insights into the pathogenic mechanisms of AS from a transcriptomic perspective. This aided in identifying potential therapeutic targets. For example, by sequencing single cells of ligament tissue, we can understand the gene expression and intercell heterogeneity of specific cells in AS patients in disease states and capture subtle gene expression changes in pathological processes such as ectopic osteogenesis, providing an important basis for early diagnosis and treatment of diseases. Despite limited information on the MLN, it remains a promising new therapeutic target for AS. The novelty of the present research lies in the integration of two-sample MR analysis with scRNA-seq validation, which provided a unique methodological approach to establishing causal relationships between circulating plasma proteins and AS. This dual approach allows for a more accurate identification of protein biomarkers and therapeutic targets, distinguishing this study from previous research that primarily relied on observational data. The findings significantly contributed to the field by not only identifying six novel protein biomarkers for AS, but also validating these biomarkers through single-cell expression analysis. This method contrasts with previous studies that have either concentrated solely on proteomics or lacked the rigorous causal inference provided by MR analysis. Comparative analysis with other studies revealed that the integrated approach of MR and scRNA-seq provided a more detailed and causally validated understanding of the role of circulating proteins in AS. This sets a new standard for future biomarker discovery and therapeutic target identification in AS and potentially other complex diseases.

However, certain limitations should be acknowledged. Firstly, our analysis focused solely on the European population, necessitating further confirmation to generalize these findings to other ancestries. Secondly, colocalization analysis did not fully mitigate potential bias from linkage imbalance, hampering the precise identification of genes with causal impacts on AS. Lastly, single-cell validation involved a small sample size and diverse racial backgrounds among patients, potentially introducing population structure bias. Therefore, future research should include larger sample sizes to address these limitations and advance our understanding of AS pathogenesis and therapeutic strategies.

## Conclusions

Our study comprehensively assessed the causal relationship between circulating plasma proteins and AS risk using two-sample MR. Our findings suggest that elevated levels of HERC5 predicted by genes are associated with increased AS risk, while the levels of the remaining five circulating proteins (AIF1, CREB3L4, MLN, MRPL55, and SPAG11B) exhibit a negative correlation with AS risk. Moreover, the differential expression of the above six protein-coding genes in AS patients was verified by single-cell sequencing. HERC5, AIF1, and MRPL55 are potential therapeutic targets for AS. In conclusion, this study’s innovative methodology and rigorous validation process provided a robust framework for identifying causally significant protein biomarkers and therapeutic targets for AS. This research not only advanced the understanding of AS pathogenesis, but also demonstrated the potential of combining proteomic MR and scRNA-seq to uncover novel insights in complex diseases.

## Supplementary Information

Below is the link to the electronic supplementary material.

Supplementary file1 (PDF 8 KB)

Supplementary file2 (PDF 12 KB)

Supplementary file3 (PDF 8 KB)

Supplementary file4 (PDF 13 KB)

Supplementary file5 (PDF 6 KB)

Supplementary file6 (PDF 7 KB)

Supplementary file7 (XLS 16441 KB)

Supplementary file8 (XLS 4211 KB)

Supplementary file9 (XLS 28 KB)

Supplementary file10 (XLS 21 KB)

Supplementary file11 (XLS 28 KB)

Supplementary file12 (XLS 22 KB)

Supplementary file13 (XLS 21 KB)

## Data Availability

The data that support the findings of this study are available on request from the corresponding author.
