# Supplementary figures and images for: Identification of novel protein biomarkers and therapeutic targets for ankylosing spondylitis using human circulating plasma proteomics and genome analysis
Source: Anal Bioanal Chem. 2024 Sep 10;416(28):6357–66. doi: 10.1007/s00216-024-05521-4 (PMC11541407; doi:10.1007/s00216-024-05521-4)

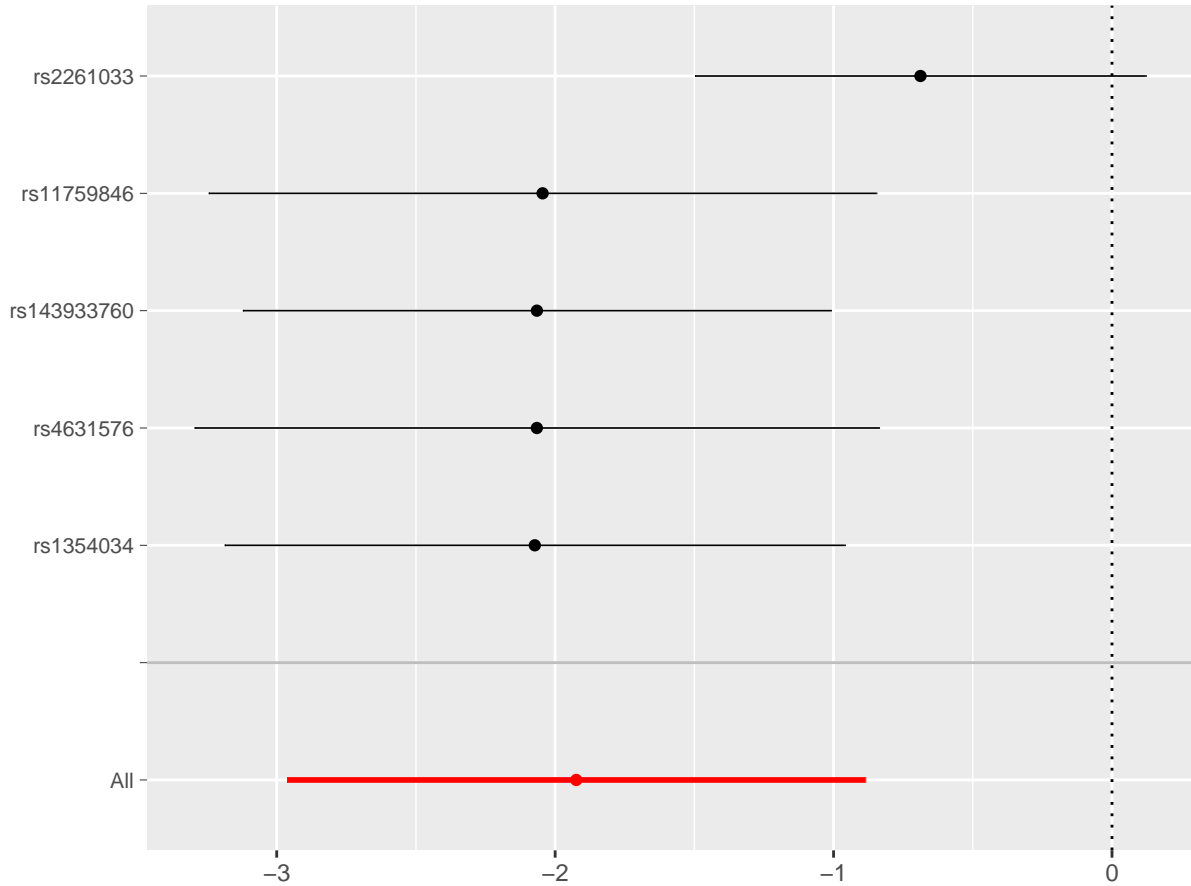

**Figure S1.** MR leave-one-out sensitivity analysis for 'AIF1' on 'Ankylosing Spondylitis'

Supplement: Supplementary file 1 — Supplementary file1 (PDF 8 KB) [file 216_2024_5521_MOESM1_ESM.pdf]

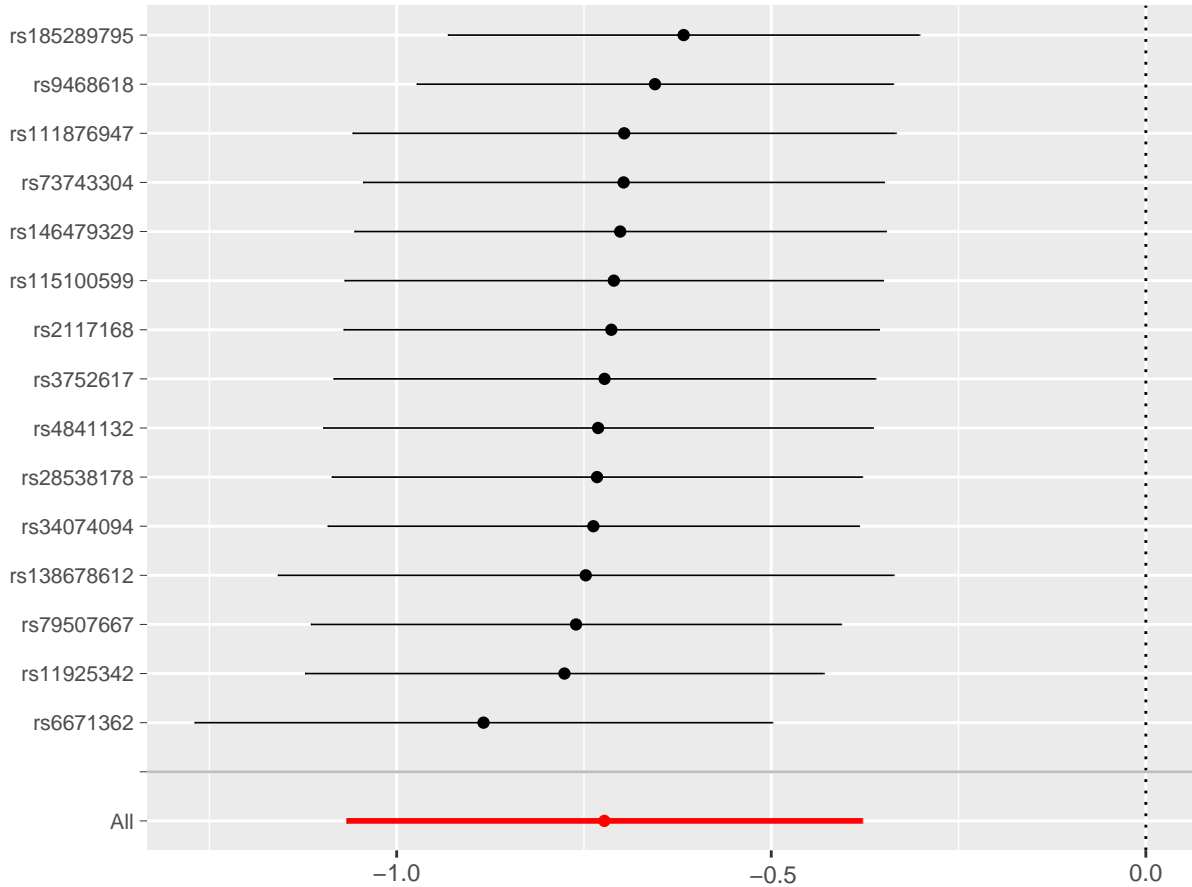

**Figure S2.** MR leave-one-out sensitivity analysis for 'CREB3L4' on 'Ankylosing Spondylitis'

Supplement: Supplementary file 2 — Supplementary file2 (PDF 12 KB) [file 216_2024_5521_MOESM2_ESM.pdf]

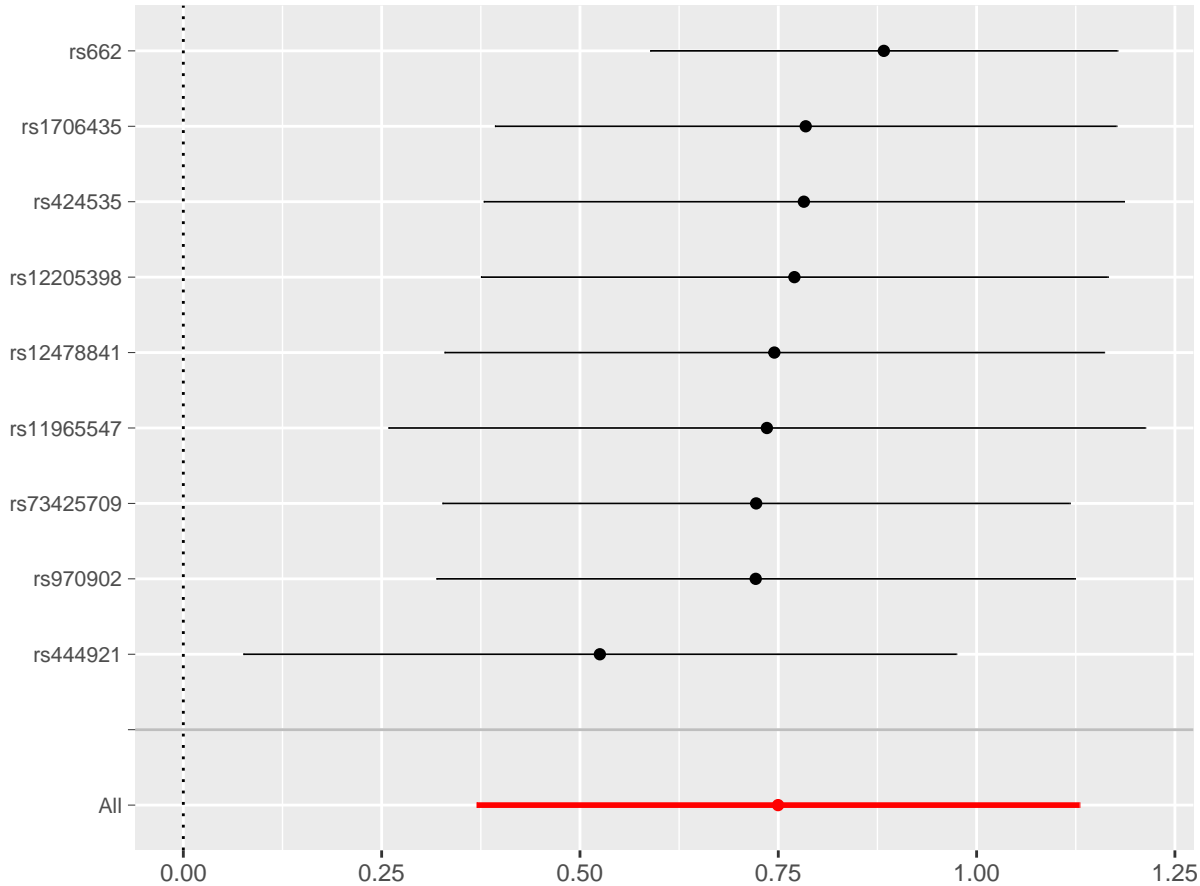

**Figure S3.** MR leave-one-out sensitivity analysis for 'HERC5' on 'Ankylosing Spondylitis'

Supplement: Supplementary file 3 — Supplementary file3 (PDF 8 KB) [file 216_2024_5521_MOESM3_ESM.pdf]

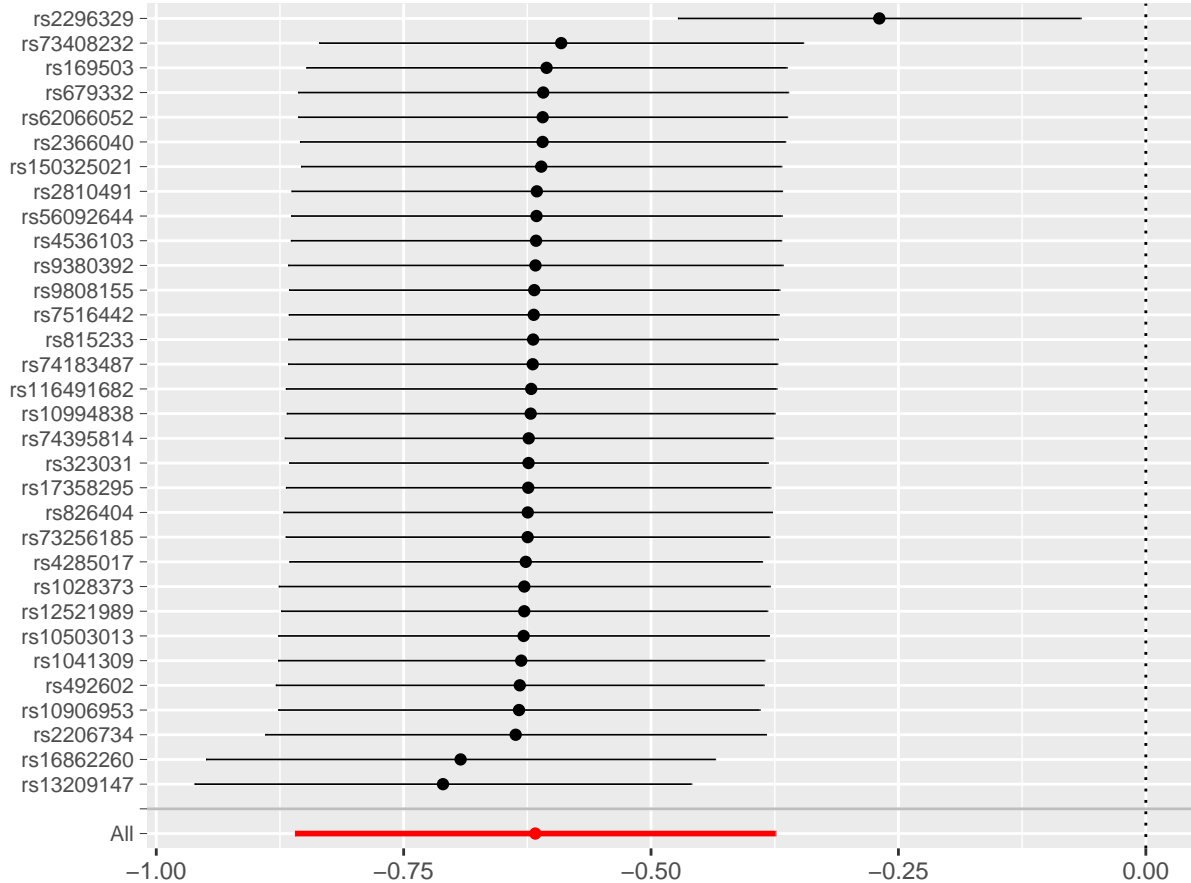

**Figure S4.** MR leave-one-out sensitivity analysis for 'MLN' on 'Ankylosing Spondylitis'

Supplement: Supplementary file 4 — Supplementary file4 (PDF 13 KB) [file 216_2024_5521_MOESM4_ESM.pdf]

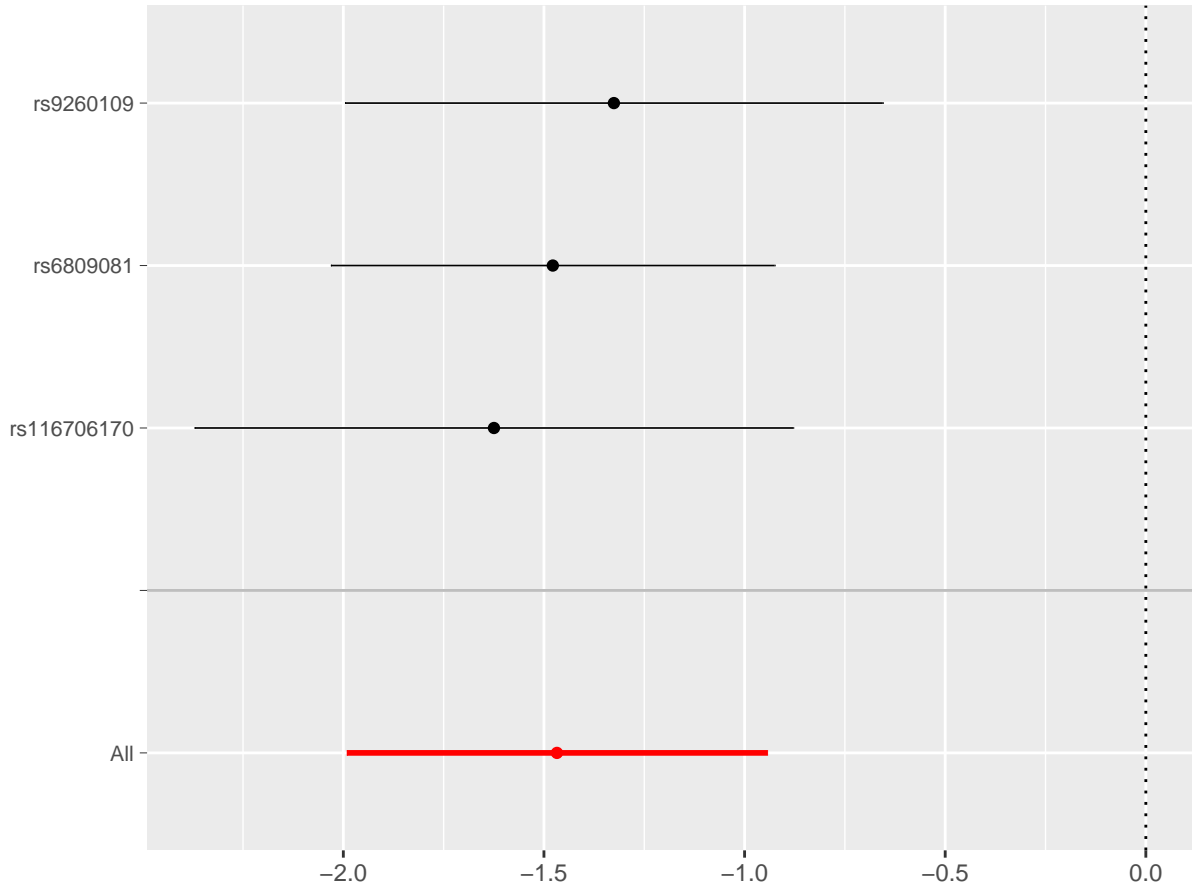

**Figure S5.** MR leave-one-out sensitivity analysis for 'MRPL55' on 'Ankylosing Spondylitis'

Supplement: Supplementary file 5 — Supplementary file5 (PDF 6 KB) [file 216_2024_5521_MOESM5_ESM.pdf]

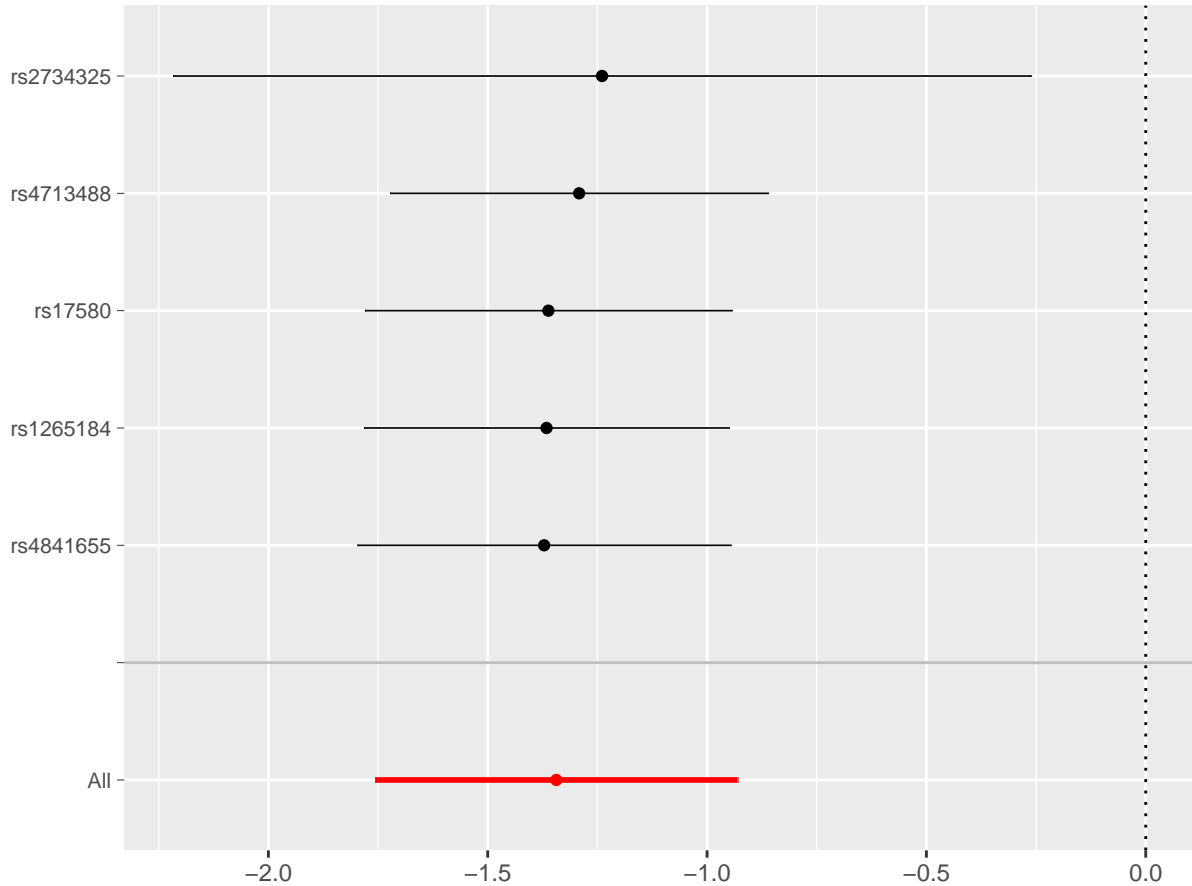

**Figure S6.** MR leave-one-out sensitivity analysis for 'SPAG11B' on 'Ankylosing Spondylitis'

Supplement: Supplementary file 6 — Supplementary file6 (PDF 7 KB) [file 216_2024_5521_MOESM6_ESM.pdf]
